# An Unusually Prolonged Case of FGF23-mediated Hypophosphatemia Secondary to Ferric Carboxymaltose Use

**DOI:** 10.1210/jcemcr/luad117

**Published:** 2023-09-27

**Authors:** Ipsa Arora, Alison Kaprove, Ronald Perrone, Lisa Ceglia

**Affiliations:** Division of Endocrinology, Tufts Medical Center, Boston, MA 02111, USA; Division of Nephrology, Tufts Medical Center, Boston, MA 02111, USA; Division of Nephrology, Tufts Medical Center, Boston, MA 02111, USA; Division of Endocrinology, Tufts Medical Center, Boston, MA 02111, USA

**Keywords:** hypophosphatemia, FGF23, ferric carboxymaltose, autosomal dominant polycystic kidney disease

## Abstract

Ferric carboxymaltose (FCM)-induced hypophosphatemia is seen in up to 75% of patients receiving this therapy for iron deficiency anemia. Hypophosphatemia has been attributed to increased circulating levels of fibroblast growth factor-23 (FGF23), the transcription of which is upregulated in an iron-deficient state. However, hypophosphatemia typically resolves within 12 weeks of FCM administration. Here, we present a case of unusually prolonged hypophosphatemia that developed after treatment with FCM in a 39-year-old female with autosomal dominant polycystic kidney disease (ADPKD) but normal renal function. Workup was significant for low tubular reabsorption of phosphate and inappropriately normal FGF23. Genetic disorders of hypophosphatemia and a FGF23-secreting tumor were ruled out. Treatment with calcitriol was required for nearly 3.5 years. The prolonged hypophosphatemia was attributed to underlying ADPKD because these patients demonstrate inappropriately elevated FGF23 levels for the degree of severity of reduced glomerular filtration rate. However, the stimulus driving FGF23 secretion in these patients is incompletely understood. Elevated FGF23 in the kidney suppresses renal tubular phosphate reabsorption and 1α-hydroxylase activity ultimately leading to hypophosphatemia. We conclude that our patient was at a high risk of developing hypophosphatemia because of underlying ADPKD, and FCM was the likely precipitant to identify this underlying process.

## Introduction

Ferric carboxymaltose (FCM) is a third-generation parenteral iron formulation commonly used to rapidly correct iron deficiency. Administration of FCM can increase circulating levels of fibroblast growth factor-23 (FGF23), an osteocyte-derived factor that promotes renal phosphate wasting by decreasing 1α-hydroxylase activity that results in reduced 1-α,25-dihydroxyvitamin D concentration and by decreasing expression of sodium phosphate cotransporters 2a and 2c, which reabsorb phosphate [1]. This increase in FGF23 has caused hypophosphatemia in the weeks after administration. According to a study by Wolf et al, hypophosphatemia was observed in 75% of patients shortly after administration of FCM, with severe hypophosphatemia (serum phosphate <2 mg/dL or <0.11 mmol/L) noted in 11.3% of cases [2]. However, FCM-associated hypophosphatemia typically resolves within 12 weeks following drug administration [3, 4]. Here, we describe a case of unusually prolonged hypophosphatemia following FCM requiring treatment for more than 3 years.

## Case Presentation

**Figure 1.**
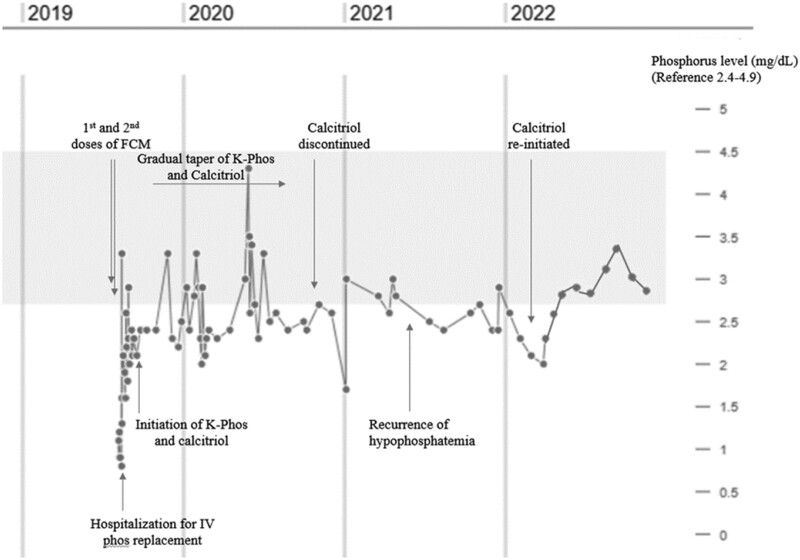
Timeline representing patient's serum phosphate levels and interventions.

A 39-year-old White female with autosomal dominant polycystic kidney disease (ADPKD), an estimated glomerular filtration rate (eGFR) of > 60 mL/min/1.73 m^2^, and celiac disease on a strict gluten-free diet, received two 750-mg doses of FCM 1 week apart by IV infusion for treatment of longstanding iron deficiency. Of note, her pretreatment levels of serum phosphate ranged between 2.7 and 2.9 mg/dL (0.15 and 0.16 mmol/L; Table 1 and Fig. 1) (reference range, 2.7-4.5 mg/dL or 0.15-0.25 mmol/L). Ferritin was 48 ng/mL (107.8 pmol/L, Table 1) (reference range, 10-240 ng/mL or 22.5-540 pmol/L). Over the ensuing 4 weeks, she developed generalized fatigue and muscle weakness with recurrent falls. She was on the vasopressin antagonist tolvaptan for ADPKD with normal serum sodium levels, and she reported no changes in medications. She denied gastrointestinal complaints. Her usual diet was high in fruit and vegetables and included protein-containing foods such as beans, nuts, and meat.

## Diagnostic Assessment

Initial biochemical testing was only notable for a new serum phosphate level of 1.1 mg/dL (0.06 mmol/L, Fig. 1). Tolvaptan was discontinued and repeat phosphate levels further reduced to 0.8 and 0.9 mg/dL (0.04 and 0.05 mmol/L). She was also noted to have normal serum electrolytes and calcium level, stable eGFR of >60 mL/min/1.73 m^2^, and a serum PTH level of 81 pg/mL (297.3 pmol/L, Table 1) (reference range, 11-95 pg/mL or 40.3-348.7 pmol/L). Given worsening phosphate levels, the patient was hospitalized to undergo IV phosphate repletion. She received 60 mmol over 2 days, which only transiently increased serum phosphate; thus, oral phosphate supplementation was initiated. Evaluation of the hypophosphatemia revealed an elevated fractional excretion of phosphate of 85% and a low tubular reabsorption of phosphate GFR of 0.9 mg/dL (Table 1). In the setting of her severe hypophosphatemia, her serum C-terminal FGF23 level was 91 RU/mL (reference range, < 180, by ELISA at Pan Laboratories, Inc.), her serum 1-α,25-dihydroxyvitamin D level was 34 pg/mL (124.8 pmol/L) (reference range, 20-70 pg/mL or 45-157.2 pmol/L), and her 25-hydroxyvitamin D was 35 ng/mL (87.5 nmol/L, Table 1). Repeat serum C-terminal FGF23 levels (ELISA, Pan Laboratories, Inc.) in the same period were even higher at 113 and 130 RU/mL (Table 1). The intact FGF23 level was 45 pg/mL (reference value, ≤59, by Chemiluminescence-Based Quantitative Sandwich Immunoassay, Mayo Clinic Laboratories, Table 1). Imaging workup was only notable for an enlarged thymus, which was felt to be unrelated. A prior dual energy x-ray absorptiometry scan revealed normal bone mineral density for age, and she denied a prior fracture history. Based on the biochemical evaluation and timing of presentation in relation to her FCM infusions, she was diagnosed with FGF23-mediated hypophosphatemia secondary to FCM.

**Table 1.** Series of events and laboratory data after ferric carboxymaltose (FCM) administration

Timeline	Phosphate, mg/dL*^a^*	TmP/GFR, mg/dL	FGF23 (c or i)*^b,c^*	PTH, pg/dL*^d^*	Calcium, mg/dL*^e^*	eGFR, mL/min/1.73 m^2^	Others*^f^*
Pre-FCM	2.7-2.9				9.3-9.5	>60	Ferritin 48 ng/mL
6/14/2019 and 6/21/2019: 2 doses of FCM 750 mg administered IV							
7/2019: Development of symptoms (fatigue, weakness, recurrent falls)							
8/12/2019 Hospitalization	0.8-0.9	0.9	91 (c)	81	9.3	>60	25(OH)D 35 ng/dL 1,25(OH)_2_D 34 pg/mL Ferritin 329 ng/mL
8/15/2019: Discharged on oral KPO_4_ (serum phosphate level, 2.1 mg/dL)							
8/23-8/26/2019 Calcitriol added	1.8-2.2		130 (c)		9.3	>60	
9/2019-2/2020 Calcitriol + KPO_4_ titration	2.0-3.3		113 (c) 45 (i)	22-41	9.1-9.8	>60	
6/2020-12/2020 Calcitriol taper (KPO_4_ stopped)	2.4-3.3			55-81	9.0-9.5	>60	Ferritin 177 ng/mL
1/2021-8/2021 Off medications	2.0-3.3	2.4		100-120	8.9-9.5	>60	25(OH)D 33 ng/dL
4/1/2022: Recurrence of severe hypophosphatemia (serum phosphate level, 1.9 mg/dL); calcitriol restarted							
5/2022-6/2023 Calcitriol only	2.7-3.1			59	8.8-9.4	50-60	

Abbreviations: cFGF23, C-terminal fibroblast growth factor-23; eGFR, estimated glomerular filtration rate; FCM, ferric carboxymaltose; iFGF23, intact fibroblast growth factor; TmP/GFR, tubular reabsorption of phosphate; 25(OH)D, 25-hydroxyvitamin D; 1,25(OH)_2_D, 1-α,25-dihydroxyvitamin D.

Reference ranges:

a 2.7-4.5 mg/dL.

b cFGF23: <180 RU/dL.

c iFGF23 <59 pg/mL.

d 11-95 pg/mL.

e 8.5-10.5 mg/dL.

f Ferritin 10-240 ng/mL, 1,25(OH)_2_D 20-70 pg/mL.

## Treatment

Treatment with oral potassium phosphate supplementation (250 mg 4 times per day) did not adequately correct serum phosphate levels. Calcitriol 0.25 mcg daily was added and later increased to twice per day. Serum phosphate levels gradually increased to 2 to 2.5 mg/dL (0.11-0.13 mmol/L); however, contrary to expectations, treatment intensified rather than lessened over the ensuing 6 months. Potassium phosphate supplements increased to 750 mg 4 times per day, causing gastrointestinal intolerance. When calcitriol was titrated up to 0.5 mcg twice per day, she was able to taper potassium phosphate to 500 mg twice per day. The patient was asymptomatic, with mildly low phosphate levels.

## Outcome and Follow-Up

Given the prolonged need for treatment, concern was raised for a possible FGF23-secreting tumor. Magnetic resonance imaging scans of the chest revealed an enlarged thymus stable in size compared with a computed tomography scan of the chest 6 months prior. An 18-gallium-DOTATATE scan did not reveal any abnormal uptake. In addition, a sequence analysis and deletion/duplication testing of 13 genes (including *FGF23*, *FGFR1*, *VDR*, *PHEX*, *CLCN5*, *DMP1*, *ENPP1*, *FAM20C, SLC34A3, CYP27B1*, and *CYP2R1*) (Invitae Hypophosphatemia Panel) were performed by Invitae Laboratory Corporation (San Francisco, CA). The results identified no significant variants in our patient.

By 12 months after FCM infusions, she was able to maintain a low normal serum phosphate level on calcitriol alone and taper from oral potassium phosphate supplementation (Table 1 and Fig. 1). Of note, following the FCM infusions, serum ferritin levels were elevated at a level of 329 ng/mL (739.2 pmol/L) at 2 months and gradually normalized to 177 ng/mL (397.7 pmol/L) by 15 months.

At 18 months, calcitriol was tapered; however, serum phosphate levels ranged slightly below the normal range between 2.2 and 2.7 mg/mL (0.12 and 0.14 mmol/L) with a rare level <2 mg/dL (0.11 mmol/L, Fig. 1). Repeat urine studies revealed a persistently low tubular reabsorption of phosphate GFR of 2.4 mg/dL (Table 1). Calcium, eGFR, and 25-hydroxyvitamin D levels were stable from previous levels, but serum intact PTH levels were mildly elevated at 100 to 120 pg/mL (367-440 pmol/L, Table 1). She denied symptoms of weakness and a repeat dual energy x-ray absorptiometry scan did not reveal any significant change in bone mineral density compared with 3 years prior. Because of her lack of symptoms and inconsistent results, she continued off calcitriol for more than a year. However, approximately 18 months after stopping calcitriol, she had a series of persistently low serum phosphate levels, with secondary hyperparathyroidism now 3 years since the FCM administration (Table 1 and Fig. 1). Following a discussion with the clinical team, the patient resumed calcitriol at 0.25 mcg daily with subsequent rapid resolution of her hypophosphatemia and hyperparathyroidism. The cause of the persistent hypophosphatemia was assumed to be mediated by FGF23, although a repeat serum level was not performed.

## Discussion

This report illustrates a case of unusually prolonged FGF23-induced hypophosphatemia precipitated by FCM administration in a patient at high risk of developing hypophosphatemia because of underlying ADPKD. The diagnosis was based on both the presentation of renal phosphate wasting in the setting of an inappropriately normal C-terminal and intact FGF23 level and the timing of presentation in relation to FCM administration. In this case, there was no evidence for a late-onset genetic cause based on testing for multiple candidate genes. There was also no evidence of a paraneoplastic cause.

The exact mechanism for FCM causing elevations in FGF23 has not been fully elucidated; however, it has been proposed that FCM, via its glucose carrier, suppresses processes involved in cleavage of the intact FGF23 by promoting O-glycosylation at Thr178 (cleavage site of iFGF23) or other posttranslational modifications that protect FGF23 from cleavage [5]. Iron deficiency anemia causes increased transcription of FGF23 [6]. When FCM is administered to patients in a state of markedly increased FGF23 transcription because of iron deficiency, even modest suppression of FGF23 cleavage might be enough to result in a rapid increase in intact FGF23 and result in severe hypophosphatemia.

Possible reasons why this case deviates from the typically reported short course of this phenomenon remain unclear but may be related to the large cumulative dose administered in a short period (2 doses of 750 mg of FCM 1 week apart) with a subsequent high ferritin level a few weeks later. A potential explanation for a milder but persistent hypophosphatemia could be related to this patient's underlying ADPKD. Patients with ADPKD, even those with chronic kidney disease stages 1 and 2 as in our patient, have 4-fold elevated levels of serum FGF23, whereas PTH, 25-hydroxyvitamin D, and 1,25-dihydroxyvitamin D levels remain in the normal range [7]. Polycystin 1 (PC1) is a protein encoded by the gene *PKD1*, mutations of which are associated with ADPKD. Interestingly, PC1 is highly expressed in osteoblasts and osteocytes, the main sources of FGF23 synthesis and secretion [8]. Conditional disruption of *PKD1* in osteoblasts leads to bone loss in mice, suggesting that PC1 regulates bone metabolism [9]. It could be hypothesized that PC1 is directly implicated in the regulation of FGF23 production, and that a genetic defect of PC1 is responsible for the increased FGF23 secretion in ADPKD [10]. Thus, we concluded that our patient was at a high risk of developing hypophosphatemia because of her underlying ADPKD and that administration of a high dose of FCM was the likely precipitant to this underlying process.

## Learning Points

- Ferric carboxymaltose (FCM) can induce hypophosphatemia because of reduced breakdown of FGF23, the transcription of which is already increased in iron deficiency anemia.
- Patients receiving FCM should be closely monitored for hypophosphatemia until there is a clear resolution. On identifying hypophosphatemia, FCM should be immediately discontinued and avoided, and an alternate iron replacement should be considered for treatment of iron deficiency.
- In cases of prolonged hypophosphatemia, patients should be evaluated for underlying bone loss. Other etiologies such as tumor-induced osteomalacia and genetic disorders should be ruled out. Treatment with calcitriol and oral phosphorus supplementation can be initiated.
- Special attention should be given to patients with underlying conditions, such as autosomal dominant polycystic kidney disease who may have high fibroblast growth factor 23 synthesis even with normal renal function.

## Contributors

All authors made individual contributions to authorship. L.C. and R.P. were involved in the diagnosis and management of this patient. I.A. was involved in data collection and manuscript submission. A.K. and R.P. were involved in reviewing and editing the manuscript. All authors reviewed and approved the final draft.

## Data Availability

Original data generated and analyzed during this study are included in this published article.

## Abbreviations

ADPKD: autosomal dominant polycystic kidney disease
eGFR: estimated glomerular filtration rate
FCM: ferric carboxymaltose
FGF23: fibroblast growth factor-23
PC1: polycystin 1
